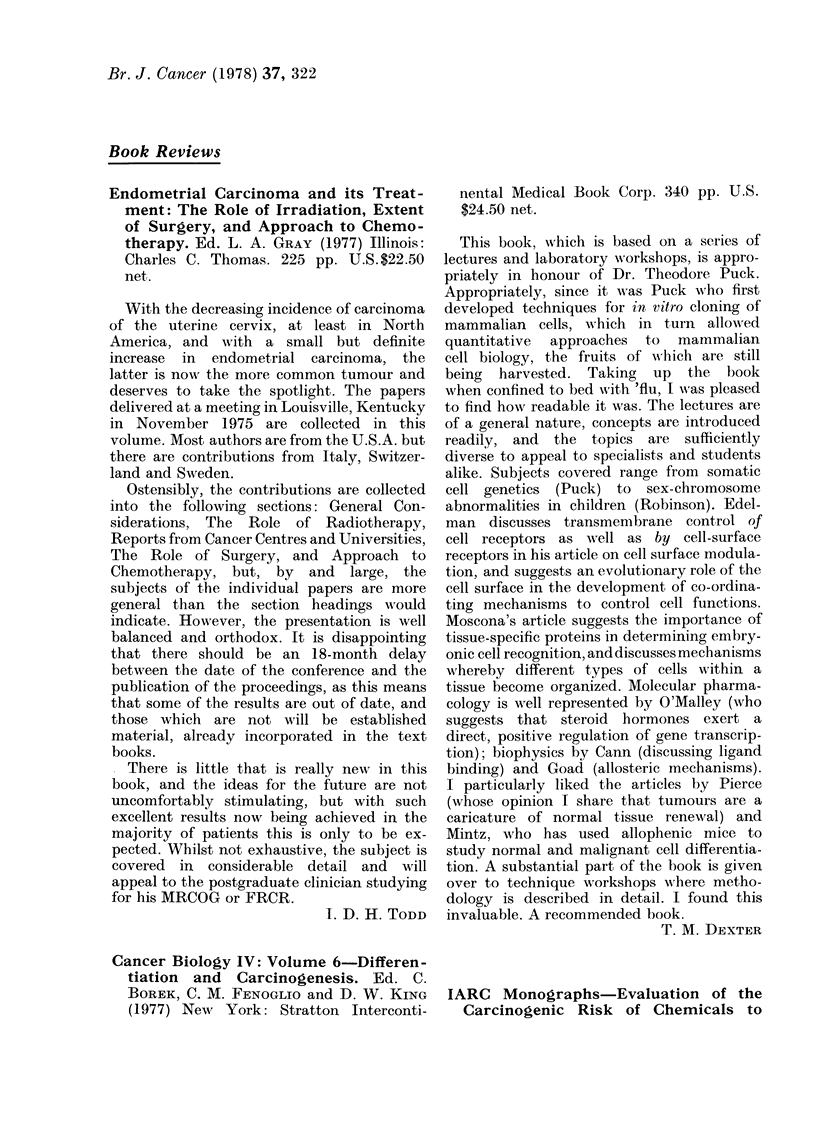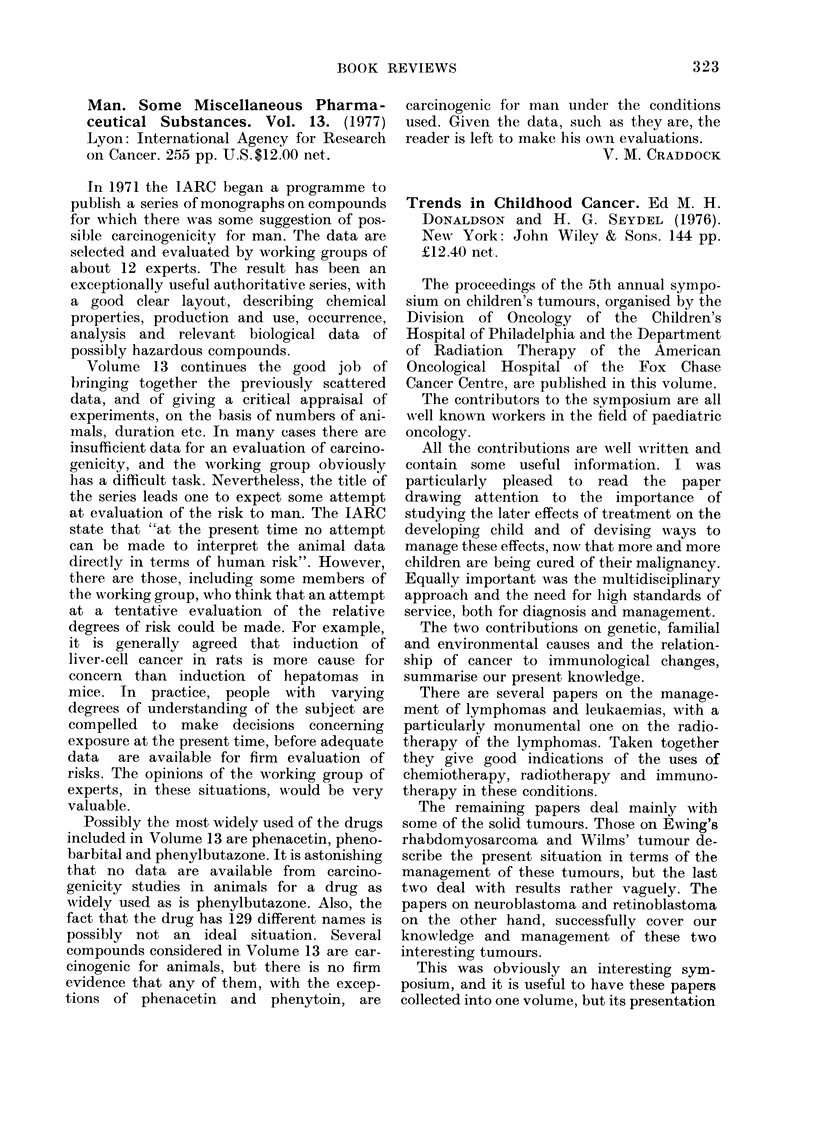# IARC Monographs—Evaluation of the Carcinogenic Risk of Chemicals to Man. Some Miscellaneous Pharmaceutical Substances. Vol. 13

**Published:** 1978-02

**Authors:** V. M. Craddock


**IARC Monographs—Evaluation of the Carcinogenic Risk of Chemicals to**

**Man. Some Miscellaneous Pharmaceutical Substances. Vol. 13.** (1977) Lyon: International Agency for Research on Cancer. 255 pp. U.S.$12.00 net.

In 1971 the IARC began a programme to publish a series of monographs on compounds for which there was some suggestion of possible carcinogenicity for man. The data are selected and evaluated by working groups of about 12 experts. The result has been an exceptionally useful authoritative series, with a good clear layout, describing chemical properties, production and use, occurrence, analysis and relevant biological data of possibly hazardous compounds.

Volume 13 continues the good job of bringing together the previously scattered data, and of giving a critical appraisal of experiments, on the basis of numbers of animals, duration etc. In many cases there are insufficient data for an evaluation of carcinogenicity, and the working group obviously has a difficult task. Nevertheless, the title of the series leads one to expect some attempt at evaluation of the risk to man. The IARC state that "at the present time no attempt can be made to interpret the animal data directly in terms of human risk". However, there are those, including some members of the working group, who think that an attempt at a tentative evaluation of the relative degrees of risk could be made. For example, it is generally agreed that induction of liver-cell cancer in rats is more cause for concern than induction of hepatomas in mice. In practice, people with varying degrees of understanding of the subject are compelled to make decisions concerning exposure at the present time, before adequate data are available for firm evaluation of risks. The opinions of the working group of experts, in these situations, would be very valuable.

Possibly the most widely used of the drugs included in Volume 13 are phenacetin, phenobarbital and phenylbutazone. It is astonishing that no data are available from carcinogenicity studies in animals for a drug as widely used as is phenylbutazone. Also, the fact that the drug has 129 different names is possibly not an ideal situation. Several compounds considered in Volume 13 are carcinogenic for animals, but there is no firm evidence that any of them, with the exceptions of phenacetin and phenytoin, are carcinogenic for man under the conditions used. Given the data, such as they are, the reader is left to make his own evaluations.

V. M. Craddock